# Association of Polygenic Risk Score and Bacterial Toxins at Screening Colonoscopy with Colorectal Cancer Progression: A Multicenter Case-Control Study

**DOI:** 10.3390/toxins13080569

**Published:** 2021-08-16

**Authors:** Alfonso Piciocchi, Elena Angela Pia Germinario, Koldo Garcia Etxebarria, Silvia Rossi, Lupe Sanchez-Mete, Barbara Porowska, Vittoria Stigliano, Paolo Trentino, Andrea Oddi, Fabio Accarpio, Gian Luca Grazi, Giovanni Bruno, Massimo Bonucci, Massimo Giambenedetti, Patrizia Spigaglia, Fabrizio Barbanti, Slawomir Owczarek, Ida Luzzi, Elisabetta Delibato, Zaira Maroccia, Lorenza Nisticò, Carla Fiorentini, Mauro D’Amato, Roberta De Angelis, Alessia Fabbri

**Affiliations:** 1 Department of Oncology and Molecular Medicine, Istituto Superiore di Sanità, 00161 Rome, Italy; alfonso.piciocchi@iss.it (A.P.); silvia.rossi@iss.it (S.R.); roberta.deangelis@iss.it (R.D.A.); 2 Department of Cardiovascular, Endocrine-Metabolic Diseases and Ageing, Istituto Superiore di Sanità, 00161 Rome, Italy; elena.germinario@iss.it (E.A.P.G.); massimo.giambenedetti@iss.it (M.G.); zaira.maroccia@iss.it (Z.M.); 3 Biodonostia, Gastrointestinal Genetics Group, Centro de Investigación Biomédica en Red de Enfermedades Hepáticas y Digestivas (CIBERehd), 20014 San Sebastian, Spain; koldo.garcia@biodonostia.org; 4 Gastroenterology and Digestive Endoscopy IRCCS Regina Elena National Cancer Institute, 00144 Rome, Italy; lupe.sanchez@ifo.gov.it (L.S.-M.); vittoria.stigliano@ifo.gov.it (V.S.); 5 Digestive Endoscopy UOC CSC03 of the Department of General Surgery, Surgical Specialities “Paride Stefanini”, Policlinic Umberto I, University of Rome ‘Sapienza’, 00161 Rome, Italy; barbara.porowska@uniroma1.it (B.P.); paolo.trentino@uniroma1.it (P.T.); fabio.accarpio@gmail.com (F.A.); 6 Hepatopancreatobiliary Surgery, IRCCS Regina Elena National Cancer Institute, 00114 Rome, Italy; andrea.oddi@ifo.gov.it (A.O.); gianluca.grazi@ifo.gov.it (G.L.G.); 7 Department of Translational and Precision Medicine, Gastroenterology Unit, Policlinic Umberto I, University of Rome ‘Sapienza’, 00161 Rome, Italy; giovanni.bruno@uniroma1.it; 8 Associazione Ricerca Terapie Oncologiche Integrate, 00165 Rome, Italy; max.bonucci@gmail.com (M.B.); carla.fiorentini@artoi.it (C.F.); 9 Department of Infectious Diseases, Istituto Superiore di Sanità, 00161 Rome, Italy; patrizia.spigaglia@iss.it (P.S.); fabrizio.barbanti@iss.it (F.B.); slawomir.owczarek@iss.it (S.O.); ida.luzzi@iss.it (I.L.); 10 Department of Food Safety, Nutrition and Veterinary Public Health, Istituto Superiore di Sanità, 00161 Rome, Italy; elisabetta.delibato@iss.it; 11 Center for Behavioral Sciences and Mental Health, Istituto Superiore di Sanità, 00161 Rome, Italy; lorenza.nistico@iss.it; 12 Gastrointestinal Genetics Lab, CIC bioGUNE, Basque Research and Technology Alliance, 48160 Derio, Spain; mauro.damato.mda@gmail.com; 13 Ikerbasque, Basque Foundation for Science, 48011 Bilbao, Spain

**Keywords:** bacterial protein toxins, colorectal cancer, gut microbiota screening, mucosa adherent bacteria, host–pathogens interaction, polygenic risk score

## Abstract

Colorectal cancer (CRC) is a leading cause of cancer death worldwide, and its incidence is correlated with infections, chronic inflammation, diet, and genetic factors. An emerging aspect is that microbial dysbiosis and chronic infections triggered by certain bacteria can be risk factors for tumor progression. Recent data suggest that certain bacterial toxins implicated in DNA attack or in proliferation, replication, and death can be risk factors for insurgence and progression of CRC. In this study, we recruited more than 300 biopsy specimens from people undergoing colonoscopy, and we analyzed to determine whether a correlation exists between the presence of bacterial genes coding for toxins possibly involved in CRC onset and progression and the different stages of CRC. We also analyzed to determine whether CRC-predisposing genetic factors could contribute to bacterial toxins response. Our results showed that CIF toxin is associated with polyps or adenomas, whereas pks+ seems to be a predisposing factor for CRC. Toxins from *Escherichia coli* as a whole have a higher incidence rate in adenocarcinoma patients compared to controls, whereas *Bacteroides fragilis* toxin does not seem to be associated with pre-cancerous nor with cancerous lesions. These results have been obtained irrespectively of the presence of CRC-risk loci.

## 1. Introduction

Colorectal cancer (CRC) is among the major threatening diseases of the present times, as in both sexes, it is the second deadliest of all cancers, responsible for more than 935,000 deaths in 2020 and the third most common cancer, with about 1,900,000 new cases diagnosed. A high mortality rate is associated with CRC and is mainly due to the delay in diagnosis, as symptoms appear late [1].

Decreasing the mortality rate by CRC is a clear, unmet need today, and the medical community agrees that the high number of CRC-related deaths could be prevented by adequate screening programs for early detection and also in order to reduce patient-care-associated costs. In the last years, it has been already proven that CRC insurgence can be considered as a multifactorial process [2]. Many studies have shown that CRC has a significant genetic basis. Moreover, alcohol consumption and tobacco smoking as well as the Westernized lifestyle, which means obesity, sedentary behavior, and high-calorie and high-fat diets, have an impact on CRC pathogenesis [3].

In addition to the environmental and genetic factors, microbial dysbiosis also influences the hallmarks of cancer [4], as does inflammation, which is strongly linked to the gut microbiota. In fact, it has been proven that pathological changes in the composition of the gut microbiota lead to intestinal inflammation and have a pivotal role in the insurgence of CRC [5]. Indeed, the long-standing presence of infection with potentially pathogenic bacteria can induce chronic inflammation, associated with CRC development in the colorectal mucosa [6]. An altered environment in the gut due to bacterial dysbiosis may lead to dysregulation of the immune system and mucus production, ultimately disrupting the delicate homeostatic relationship between commensal bacteria and the human host [7]. Thus, while the causes of CRC are not completely established, it is becoming increasingly clear that the gut microbiota provides a crucial contribution. An emerging aspect is that chronic infections triggered by certain bacteria can be risk factors for tumor progression [8]. In this context, recent studies report an increase in the abundance of *Fusobacterium* in human CRC compared to controls, suggesting an association with the later stages of CRC [9]. Although a great number of studies have focused on how dysbiosis leads to CRC, the complex role of bacterial toxins in cancer insurgence and progression is emerging as a significant topic that deserves considerable attention. In fact, pathogenic bacteria primarily use their toxin-mediated assault strategies to create a favorable host cell environment. However, their toxins can exert a pro-tumoral activity in multiple ways, such as host cell DNA damage and induction of genomic instability, cell death resistance, signaling involved in cell proliferation, and inflammation [10,11]. The strongest evidence is for *Helicobacter pylori* CagA, a protein that activates the pro-oncogenic catenin and induces an invasive phenotype [12], and for enterotoxigenic strains of *Bacteroides fragilis* (ETBF), known to produce the *B. fragilis* toxin (BFT), whose long-term colonization may increase CRC risk [13]. Several studies have been conducted in recent years highlighting that bacterial toxins could induce hallmarks of cancer via two main routes. One route is a direct attack to DNA that causes mutations and genome instability, as in the case of the genotoxin colibactin, produced by *Escherichia coli* strains and positive for the genomic island polyketide synthetase (pks), or the cytolethal distending toxin (CDT), also produced by *E. coli*. The other route is an engagement of signaling pathways that modulate cell proliferation, replication, and death, ultimately resulting in transformation, as for BFT and the *E. coli* cytotoxic necrotizing factor 1 (CNF1) or the cycle-inhibiting factor (CIF) (for an updated review, see Fiorentini et al. [11]). In this context, an exciting recent finding strongly supports the correlation between bacterial toxins and cancer since it has been discovered that mutations detected in CRC match the gut bacterium pks+ *E. coli* signature [14].

Inherited genetic factors underlie ~30% of all cases of CRC, but the high-penetrance germline mutations in known genes are responsible for only <5% of cases [15], with much of the variation in genetic risk likely to be a consequence of common genetic variations detectable through genome-wide association studies (GWAS) [16]. Whether a synergy exists between the presence of bacterial toxins and predisposing genetic factors is a still completely unexplored area.

In the present study, we recruited more than 300 biopsy specimens from people undergoing colonoscopy to analyze whether a correlation exists between the presence of bacterial genes coding for toxins (CDT, CIF, CNF1, BFT, and colibactin-positivity for the genomic island pks) and the different stages of CRC. Moreover, we aimed analyze the possibility that known CRC-risk loci contribute to the response to bacterial toxins. For this purpose, we created a database with patients’ information, and we explored the association of the bacterial toxins with hyperplastic polyps (HP), pre-cancerous polyps/adenomas (PA), and adenocarcinomas (ADK).

## 2. Results

Colorectal biopsies from 330 eligible individuals were analyzed. We excluded samples from five subjects because of vials A and B inversion. Samples from the remaining 325 subjects, 162 men and 163 women, included healthy tissues (162, controls) or tissues with hyperplastic polyps (55, HP), pre-cancerous polyps or adenomas (79, PA), or adenocarcinomas (29, ADK), as shown in Table 1.

ADK patients were more frequent in Regina Elena National Cancer Institute (RE, Roma, Italy) (83%), while HP patients were mostly collected by Policlinico Umberto I Sapienza University (SU, Roma, Italy) (82%). Female cases were 45% (13) in the ADK group and 59% (96) in controls.

The median age of enrolled subjects was 60 years, with higher values for ADK patients (68 years) compared to PA (60 years), HP (62 years), and controls (59 years). BMI ranged from 13.8 to 40.4 (median at 24.9). Information on alcohol (spirits) and wine consumption, type of diet, smoking condition, and physical activity was highly complete (>95%). Insufficient data quality and high proportion of missing values were observed for meat consumption (70%, not shown). Previous removal of polyps was reported for 130 subjects (40%) and was the prevalent condition of patients with HP (60%). Overall, 89% of the biopsies were sampled from the colon and 11% from the rectum. Sigma was the prevalent location for controls (75%), HP (40%), and PA (27%), while ADK was more frequently diagnosed in the rectum (45%).

**Figure 1:**
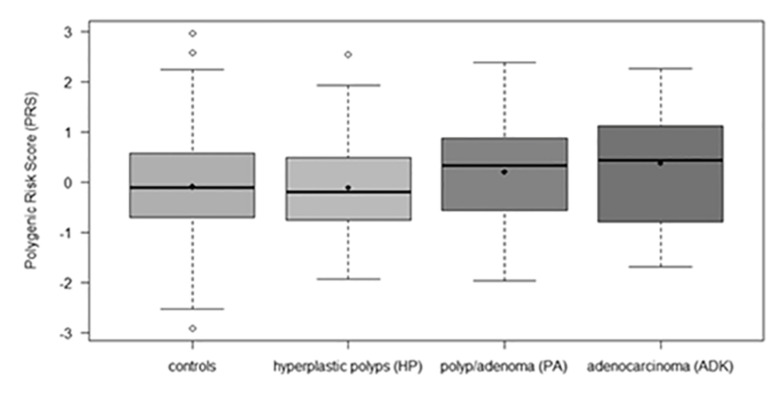
Box plot of Polygenic Risk Score by outcome measures.

As depicted in Figure 1, the Polygenic Risk Score (PRS) was similar between controls and HP patients and increased with cancer progression (PA and ADK): median PRS was 0.43 for ADK patients, 0.33 for PA, and −0.11 for controls (*p*-value for trend: 0.0124).

The overall and outcome-specific distribution of the bacterial toxins is shown in Table 2.

Among aerobic bacteria, the most frequent trait in the whole population was the positivity for the pks island (pks+) (117, 38%), followed by *cnf1* (29%), *cdt* (13%), and *cif* (10%).

In the PA group, *cif* (16% vs. 7%) and *cdt* (20% vs. 12%) were detected more frequently compared to controls, while pks+ and *cnf1* incidence rate was higher in ADK patients, 52% and 41%, respectively, compared to controls (35% and 27%). *cdt* incidence was similar in the ADK e PA group, 20% vs. 19%, respectively.

Overall, the *bft* toxin gene emerged in only 43 samples (13%). *bft*-positivity rate was similar in all outcome types, ranging from 11% in HP cases to 14% in both controls and ADK groups.

### 2.1. ADK vs. Controls Analysis

Factors associated within 10% tolerance (*p*-value < 0.1) with ADK in univariate logistic regression models were represented by the presence of pks+, sum of aerobic bacterial toxins, PRS, and age, as shown in Table 3. Other parameters had no significant association with ADK. PRS represented the only adverse parameter in both univariate and multivariate analysis (OR: 1.55, 95% CI: 1.00–2.45, *p-*value: 0.055).

### 2.2. PA vs. Controls Analysis

The presence of *cif* was significantly associated with pre-cancerous PA in univariate analysis. Other significant factors within 10% tolerance were PRS and sex, as reported in Table 4.

In the multivariate logistic regression model for PA condition, after adjusting for all parameters with univariate association, *cif* toxin gene (OR: 2.57, 95% CI: 1.06–6.33, *p*-value: 0.037) and sex (F vs. M OR: 0.48, 95% CI: 0.28–0.85, *p*-value: 0.011) were the only risk factors that retained statistical significance.

### 2.3. HP vs. Controls Analysis

BMI (OR: 1.10, 95% CI: 1.01–1.19, *p*-value: 0.026), non-drinker condition (OR: 0.23, 95%CI: 0.05–0.73, *p*-value: 0.026), and previous benign vs. malignant removal of polyps (OR: 5.45, 95% CI: 1.90–18.3, *p*-value: 0.003) resulted to be independent risk factors for HP condition in the multivariate logistic regression analysis applied to models, including risk factors proven to be significant in the univariate analysis. Factors no more significant after adjustment in multivariate analysis were sex, alcohol consumption, and smoking (Table 5).

## 3. Discussion

Bacterial dysbiosis is critically involved in CRC and recent reports suggest that some toxins produced by pathogenic bacteria can play a role in CRC onset and progression. However, a clear demonstration of a correlation between the presence of bacterial toxins and CRC is still missing. The present study was designed to evaluate whether detection of bacterial toxins genes in colorectal tissue was positively associated with pre-cancerous or cancerous lesions and whether known CRC-predisposing genetic factors could contribute to the response to bacterial toxins. Our results indicate that the *cif* toxin gene is significantly associated with pre-cancerous PA of colon-rectum compared to healthy tissues. On the other hand, the *E. coli* toxins as a whole seem to have a higher incidence rate in ADK patients compared to controls, whereas pks+ seems to be a predisposing factor for CRC. By contrast, *bft* from *B. fragilis* resulted not to be associated neither with pre-cancerous lesions nor with cancerous ones. The sample size was sufficient to confirm the etiological role of the selected PRS for CRC [17]. In particular, PRS was similar between controls and HP patients and increased with cancer progression. Moreover, despite the limited CRC sample, PRS resulted as the risk factor more robustly associated with ADK after multivariable adjustment. It is worth noting that, since the model is based on a genetic study of CRC, the PRS works better in ADK rather than in PA.

To our knowledge, this is the first study dealing with a high number of mucosa samples, in particular for pre-cancerous lesions. We obtained only a reduced sample size of cancerous lesions. This fact implies that our observations for pks+ in relation to ADK are not statistically significant, but a trend that encourages further studies was nonetheless revealed.

Our results clearly indicate that the *cif* gene is positively associated with pre-cancerous lesions, and it is the only risk factor that retained statistical significance after multivariate analysis. Interestingly, CIF is the less studied among toxins potentially linked to CRC. To our knowledge, the only paper considering CIF in this context is by Buc and coworkers [18], who demonstrated that there is a high prevalence of cyclomodulin-producing *E. coli* in biopsies of CRC, cyclomodulins being represented by CDT, colibactin (encoded by pks region), CIF, and CNF1. CIF acts by deamidating NEDD8 proteins [19], ultimately preventing ubiquitylation of a number of substrates and thus blocking their degradation by the 26S proteasome [20,21,22]. By blocking the degradation of proteins, CIF modifies a number of activities in cells. In fact, it enhances virulence primarily by abolishing the bactericidal activity of Perforin-2 [23] and mediates the inflammatory tolerance of commensal bacteria by preventing IkB and β-catenin degradation [24,25]. In addition, CIF accumulates p21^Waf1/Cip1^ and p27^kip2^ proteins, thus arresting the cell cycle at the G2/M or at the G1/S transition [20,22,26] depending on the cellular-cycle phase at the moment of infection. CIF-mediated cell-cycle arrest might slow down epithelial cells’ renewal, thus providing enteric pathogens with a stable platform that favour gut colonization [27]. For its action on the cell cycle, CIF was included in the so-called cyclomodulins, and together with other toxins somehow touching the cell cycle, it was indicated as a putative toxin implied in CRC [28].

Although not statistically significant, our results, showing that *E. coli* toxins genes as a whole seem to have a higher incidence rate in ADK patients, are in agreement with previous studies. In particular, *E. coli* are commonly isolated from both CRC patients and healthy controls. However, in CRC patients, more pathogenic *E. coli* strains are found [29,30]. In this context, Buc and coworkers [18] reported that there is a high prevalence of cyclomodulin-producing *E. coli* in biopsies of CRC. The presence of the genomic island pks+ in particular seems to be a predisposing factor for CRC. pks+ is one of the most studied factors related to CRC development, and several groups reported that mucosa samples from CRC patients more frequently harbour pks+ *E. coli* strains with respect to healthy patients [18,31,32].

The fact that the product of the genomic island pks+ (the so-called colibactin) can be considered a predisposing factor for CRC is also supported by its activity in eukaryotic cells since it induces DNA damage, cell-cycle arrest, mutations, and chromosomal instability [33,34,35,36], all events known to contribute to tumorigenesis. Very recently, in a human intestinal organoid affected by prolonged exposure to *E. coli* expressing pks+, the same mutational signature detected in a subset of human cancer genomes was found [14], further indicating a possible role for the product of the genomic island pks+, colibactin, in CRC. We would like to underline that the other two *E. coli* toxins genes herein studied, *cnf1* and *cdt*, did not reach any significant association with HP or PA alone. However, due to the low number of ADK, we cannot rule out the possibility that they can be associated with cancerous lesions. Both of them, in fact, are closely studied for their activities strongly reminiscent of carcinogenesis [11,37,38].

As regards *B. fragilis*, our results revealed no association between the presence of *bft* and HP and PA and ADK, highlighting a discrepancy with previous reports from other groups. *B. fragilis* is a normal inhabitant of the gastrointestinal tract, but the enterotoxigenic form producing BFT is present in approximately 20% of the healthy population [39]. Current evidence suggests that BFT may be a driver for chronic colitis and CRC [40,41], and its mode of action strongly supports this role. BFT, in fact, is a metalloprotease that cleaves the extracellular domain of the tumor suppressor protein E-cadherin, inducing a number of events, such as migration of β-catenin to the nucleus, activation of the c-Myc pathway, induction of MAPKs and the NF-κB pathway, and secretion of chemokines, which ultimately lead to loss of cell–cell contacts, cell rounding, and proliferation [42,43]. In animal models, *B. fragilis* strains producing BFT have been shown to contribute to carcinogenesis [44,45,46], actively supporting BFT as a CRC inducer. In addition, different reports have shown a significant association between the *bft* gene and pre-cancerous and cancerous lesions in human [47,48,49] Thus, it seems that our results on *bft* are not in agreement with the current literature. We have to underline, however, that studies revealing an association between *bft* and CRC strongly differ from our study in terms of sample dimension, exclusion criteria, and statistical analyses. In fact, no more than 60 cases and 60 controls were analysed in a single study; univariate analysis was only applied, and stool samples instead of mucosa were used in some cases. Moreover, due to the low number of ADK samples in our study, we cannot exclude the existence of an association between *bft* and cancerous lesions.

## 4. Conclusions

Our results indicate for the first time a role for the CIF toxin in the early stages of carcinogenesis and pave the way for further insights into the association of *E. coli* toxins and BFT with cancerous lesions, with the aim of exploring the possibility of incorporating bacterial toxins as biomarkers into CRC screening protocols or as tools for risk stratification.

## 5. Materials and Methods

An observational case-control study was designed to evaluate if the presence of bacterial toxins (one or more) in colorectal tissue is positively associated with pre-cancerous (PA) or cancerous lesions (ADK). Colorectal biopsies with polyps/cancer (cases) were compared to disease-free colorectal tissues (controls). Moreover, the study evaluated whether known CRC-predisposing genetic factors contribute to the response to bacterial toxins.

### 5.1. Study Design, Ethics Procedures and Adherence to STROBE Guidelines

The research was conceived as a collaborative study among Istituto Superiore di Sanità (ISS, Roma, Italy), Policlinico Umberto I Sapienza University, Regina Elena National Cancer Institute, and Karolinska Institutet. It was approved by the ISS Ethical Committee (reference number: PRE 564/16) and carried out in accordance with the recommendations, with written informed consent from all subjects. All subjects involved were adult and gave written, informed consent in accordance with the Declaration of Helsinki.

The Strengthening the Reporting of Observational Studies in Epidemiology (STROBE) guidelines were followed in processing this study [50].

### 5.2. Enrolment of Patients and Selection of Study Subjects

Intestinal biopsy samples were obtained from 330 Caucasian adult subjects that underwent colonoscopy between September 2016 and July 2018.

**Figure 2:**
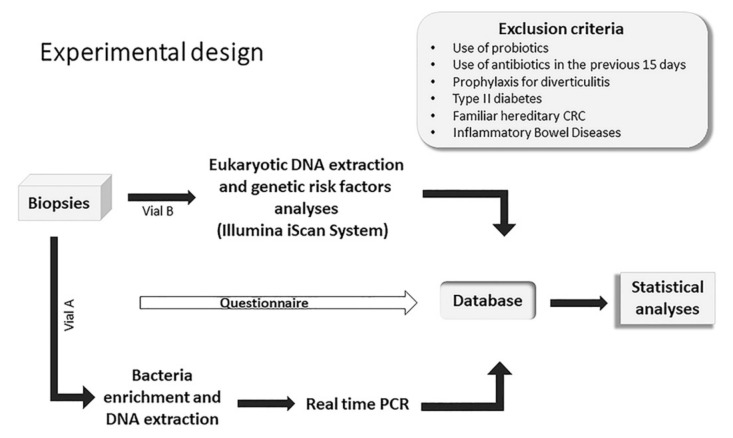
Experimental design and exclusion criteria of the study.

Before the procedures, a questionnaire was administered to collect information on subjects’ anamnesis and lifestyle. Exclusion criteria included the use of probiotics and antibiotics in the previous 15 days; prophylaxis for diverticulosis; type II diabetes; familiar hereditary CRC and inflammatory bowel diseases. A database of the subjects enrolled was created where age, sex, clinical history, date of sampling, histopathology examination, information on the nature of the polyp (benign or transformed), as well as the presence of the bacterial toxins gene and the genetic risk factors obtained were specified. The experimental design as well as exclusion criteria are reported in Figure 2 and further explained below.

### 5.3. Biopsy Sample Collection and Storage

All the patients received polyethylene glycol as sole bowel preparation for colonoscopy. During the procedure, biopsies were taken from the normal-appearing colic mucosa as well as from HP, PA, ADK, or portions of the colon immediately adjacent to these lesions. For each patient, two biopsies were taken, and each one was put in a different vial: the first one at or near the lesion (Vial A) and the second one far from the lesion (Vial B). For controls, the two biopsies were taken in the same place. The biopsy in Vial A was used for bacteria enrichment and real-time PCR to detect the presence of bacterial toxins genes. The biopsy in Vial B was used for genotyping of CRC-risk loci and was taken far from the lesion to avoid mutations due to the lesion itself. Collected biopsies were rapidly frozen in dry ice and kept frozen until subsequent use.

### 5.4. Bacteria Enrichment and DNA Extraction

Biopsies were grown on appropriate media to enrich aerobic and anaerobic bacteria. In particular, for aerobic bacteria, each bioptic fragment was placed in 3 mL Trypticase Soy Broth (TSB, Becton Dickinson GmbH, Heidelberg, Germany) tube. After overnight incubation at 37 °C under aerobic condition, 1 mL of TSB culture was centrifuged at 10,000× rpm for 10 min, and the bacterial pellets were kept frozen until subsequent use. For anaerobic bacteria, i.e., *B. fragilis*, biopsy specimens were incubated at 35 °C in anaerobic conditions (90% N_2_, 5% H_2_, 5% CO_2_) in 10 mL of Brain Heart Infusion broth (Oxoid) supplemented with 1 g/L of cysteine, 5 mg/L of hemin solution, 20 mL/L of 10% NaHCO_3_ solution, and 100 mg/L of gentamycin. After 48 h of growth, 1 mL of culture broth was centrifuged at 10,000× rpm for 3 min, and pellets were kept frozen until subsequent use. To extract DNA, pellets from aerobic or anaerobic cultures were lysed by adding 200 μL of sterile water, vortexing, and heating at 100 °C for 10 min. Following centrifugation at 14,000× rpm for 5 min, DNA-containing supernatants were transferred to sterile tubes and kept frozen until subsequent analyses.

### 5.5. Real-Time PCR Analysis

Real-time PCR amplification was performed on total bacterial DNA obtained from colon biopsies cultured in aerobiosis or anaerobiosis, as previously described, in order to detect the presence of genes for the virulence factors under study. The presence of *gapA* and *bfra* genes was used as control for the presence of aerobic bacteria or *B. fragilis*, respectively.

Amplification reactions were performed with Bio-Rad CFX96 platform, using a 96-well PCR multiplate from Bio-Rad. Samples were analyzed by means of SYBR Green Mastermix (Qiagen QuantiTect SYBR Green PCR Kit (1000) or Bio-Rad SsoAdvancedTM Universal SYBR Green Supermix). As positive controls, we used *E. coli* strain EF4 for *gapA*, *cdt,* and *cif*; *E. coli* strain J96 for *cnf1*; *E. coli* Nissle 1917 for *clbA* and *clbQ* (pks+); and *B. fragilis* for *bfra* and *bft*.

Amplification conditions were optimized for each target used (*cif*, *cnf1*, *gapA*, *bfra*, *bft*, *clbA*, *clbQ,* and *cdt*) to obtain a specific melting curve for each amplicon.

In Table S1, the primers for each molecular target and the condition used are summarized.

The thermal profile using QuantiTect SYBR Green PCR Kit (Qiagen, Hilden, Germany) for the amplification of *gapA*, *cnf1*, *cif*, *cdt*, *bfra,* and *bft* was performed using an initial hot-start step at 95 °C for 15 min, followed by 39 cycles at 94 °C for 30 s, 52 °C for 1 min, 72 °C for 1 min, 95 °C for 10 s, and 65 °C for 5 s. The thermal profile for the amplification of *clbA* and *clbQ* (both necessary to determine pks+) using SsoAdvancedTM Universal SYBR Green Supermix (Bio-Rad) was 95 °C for 5 min, followed by 39 cycles at 95 °C for 30 s, 55 °C for 1 min, 72 °C for 1 min, 95 °C for 10 s, and 55 °C for 5 s. The fluorescence measurements were followed on-line by the software of the instrument. Two PCR replicates were carried out for each standard solution and control.

### 5.6. DNA Extraction and Genotyping of CRC Risk Loci

Total DNA was extracted from biopsies by using the QIAamp Fast DNA tissue kit (Qiagen, Hilden, Germany), according to the manufacturer’s instructions. This kit uses a combination of mechanical, chemical, and enzymatic lysis to homogenize samples. Briefly, biopsies were destroyed by mechanical agitation in tubes containing beads. Homogenization of the tissue and simultaneous stabilization of DNA released from the disrupted tissue was obtained by optimized chemistry. Digestion with a buffer containing proteinase K completed the lysis of samples, and genomic DNA was subsequently purified using the QIAamp Mini Spin Columns provided.

DNA samples were genotyped using Illumina Global Screening Array on Illumina iScan high-throughput screening system in the Institute of Clinical Molecular Biology (Kiel, Germany). The raw-intensity data were analyzed using the GenCall algorithm available in the software GenomeStudio to call the alleles.

Genotypes were QCed, removing samples and markers with the following criteria: exclusion of samples with ≥15% missing rates; exclusion of markers with non-called alleles; exclusion of markers with missing call rates > 0.05; exclusion of samples with ≥5% missing rates; exclusion of related samples (PI-HAT > 0.1875); exclusion of samples whose genotyped sex could not be determined; exclusion of samples with high heterozygosity rate (more than 3 times standard deviation from mean); removal of SNPs (Single Nucleotide Polymorphism) located in sexual chromosomes; removal of markers with Hardy–Weinberg equilibrium *p*-value < 1 × 10^−5^; removal of markers whose *p*-value of difference in missingness between adenoma and colorectal cancer cases and healthy controls was <1 × 10^−5^; and removal of samples that were outliers, identified using principal component analysis (deviation of more than 6 times interquartile range). A total of 279 samples were kept after the QC.

SNPs not genotyped were imputed by means of Sanger Imputation Server. The release 1.1 of Haplotype Reference Consortium was used as reference panel, and the imputation was done using the EAGLE2 + PBWT pipeline [17,51,52,53].

To calculate the Polygenic Risk Score, the study GCST007856 [17] deposited in GWAS Catalog was used as model, and PRSice2 software [54] was used to derivate the score of each sample.

### 5.7. Statistical Methods

The sample size was planned to assume a three-fold prevalence of bacterial virulence factors genes in pre-cancerous PA compared to controls (A.F.’s preliminary data). A sample size of 75 cases and 150 controls was estimated to be sufficient to assess the association with an 80% power.

Subjects’ characteristics for the whole population and stratified by outcome (controls, HP, PA, and ADK) were summarized by means of the levels of the variables (with%) for categorical variables or by means of quantiles and mean with standard deviation for continuous variables.

Potential exposure factors were represented by the presence of bacterial toxins genes (*cif*, *cdt*, *cnf1*, pks+, *bft*). The role of bacterial toxins genes on the outcome was also analyzed considering the sum of aerobic toxins and the sum of all toxins.

Factors used to describe the sample and to adjust the effect of bacterial toxins on the outcome were PRS, age, sex, smoking, alcohol consumption, body mass index (BMI), physical activity, and other health status and lifestyle variables.

Compliance with the study was evaluated in terms of percentage of missing values. Variables with high level of missing values were considered only for descriptive purposes; no imputation methods were used.

In univariate analysis, non-parametric tests were performed for comparisons between groups (chi-square and Fisher’s exact test in case of categorical variables or response rate; Mann–Whitney and Kruskal–Wallis test in case of continuous variables). Logistic regression models were used in univariate and multivariate analyses to assess if the presence of bacterial toxins (one or more) in colorectal tissue was positively associated with PA or ADK compared to disease-free colorectal tissues. Odds ratios and 95% confidence interval were reported as parameter results of the logistic regression models. All covariates were evaluated in univariate models, and all factors with univariate association within *p*-value < 0.1 were considered in the multivariate models. Backward and stepwise methods were applied to identify the multivariate models with a step-by-step iterative construction that involved the selection of independent variables to be considered in the final model.

All tests were 2-sided, accepting *p*-value < 0.05 as indicating a statistically significant difference, and CI were calculated at 95% level. All analyses were performed using the R software [55]. Logistic regression models were fit using the R glm function with a binomial distribution and a logit link function.

## Figures and Tables

**Table 1:** Overall and outcome-specific distribution of the covariates analyzed in the study protocol.

Characteristic	Overall, N = 325	Healthy Tissue (Controls), N = 162	Hyperplastic Polyps (HP), N = 55	Polyps/Adenoma (PA), N = 79	Adenocarcinoma (ADK), N = 29
**Hospital, n (%)**					
*SU*	184 (57%)	80 (49%)	45 (82%)	54 (68%)	5 (17%)
*RE*	141 (43%)	82 (51%)	10 (18%)	25 (32%)	24 (83%)
**Sex, n (%)**					
*M*	162 (50%)	66 (41%)	34 (62%)	46 (58%)	16 (55%)
*F*	163 (50%)	96 (59%)	21 (38%)	33 (42%)	13 (45%)
**Age**					
*Mean (SD)*	60 (12)	59 (12)	60 (11)	61 (12)	64 (14)
*Median (range)*	60 (22, 87)	59 (22, 87)	62 (29, 81)	60 (36, 87)	68 (34, 85)
**BMI**					
*Mean (SD)*	25.4 (3.9)	24.9 (4.1)	26.1 (4.1)	25.8 (3.4)	25.9 (3.9)
*Median (range)*	24.9 (13.8, 40.4)	24.2 (13.8, 40.4)	26.1 (16.4, 37.4)	25.7 (19.7, 37.5)	25.8 (19.4, 31.8)
*Unknown*	6	5	0	0	1
**Alcohol consumption, n (%)**	76 (23%)	35 (22%)	20 (36%)	16 (20%)	5 (17%)
**Wine consumption, n (%)**	165 (51%)	75 (46%)	31 (56%)	45 (58%)	14 (48%)
*Unknown*	1	0	0	1	0
**Physical activity n (%)**	129 (40%)	65 (40%)	24 (44%)	32 (41%)	8 (29%)
*Unknown*	2	1	0	0	1
**Non-drinker, n (%)**	52 (16%)	30 (19%)	3 (5.5%)	10 (13%)	9 (32%)
*Unknown*	3	2	0	0	1
**Diet, n (%)**					
*Mediterranean*	293 (92%)	148 (93%)	45 (82%)	73 (99%)	27 (93%)
*Vegetarian*	15 (4.7%)	8 (5.0%)	5 (9.1%)	0 (0%)	2 (6.9%)
*Vegan*	1 (0.3%)	1 (0.6%)	0 (0%)	0 (0%)	0 (0%)
*Other*	8 (2.5%)	2 (1.3%)	5 (9.1%)	1 (1.4%)	0 (0%)
*Unknown*	8	3	0	5	0
**Smoking, n (%)**					
*No*	153 (47%)	80 (49%)	18 (33%)	43 (54%)	12 (41%)
*Yes*	68 (21%)	30 (19%)	19 (35%)	15 (19%)	4 (14%)
*Ex*	104 (32%)	52 (32%)	18 (33%)	21 (27%)	13 (45%)
**Previous removal of polyps, n (%)**					
*Malignant polyp*	50 (15%)	30 (19%)	5 (9.1%)	11 (14%)	4 (14%)
*Benign polyp*	80 (25%)	29 (18%)	28 (51%)	21 (27%)	2 (7.1%)
*No removal*	191 (59%)	101 (63%)	22 (40%)	46 (59%)	22 (79%)
*Unknown*	4	2	0	1	1
**Biopsy site, n (%)**					
*Caecum*	25 (7.7%)	5 (3.1%)	4 (7.3%)	14 (18%)	2 (6.9%)
*Ascending*	54 (17%)	27 (17%)	9 (16%)	17 (22%)	1 (3.4%)
*Hepatic flexure*	5 (1.5%)	0 (0%)	1 (1.8%)	4 (5.1%)	0 (0%)
*Transverse*	13 (4.0%)	1 (0.6%)	6 (11%)	6 (7.6%)	0 (0%)
*Descending*	15 (4.6%)	0 (0%)	2 (3.6%)	11 (14%)	2 (6.9%)
*Sigma*	174 (54%)	120 (75%)	22 (40%)	21 (27%)	11 (38%)
*Colon nas*	1 (0.3%)	1 (0.6%)	0 (0%)	0 (0%)	0 (0%)
*Rectum*	37 (11%)	7 (4.3%)	11 (20%)	6 (7.6%)	13 (45%)
*Unknown*	1	1	0	0	0
**Polygenic Risk Score (PRS)**					
*Mean (SD)*	0.01 (1.00)	−0.10 (1.01)	−0.11 (0.95)	0.20 (0.94)	0.36 (1.15)
*Median (range)*	0.03 (−2.92, 2.96)	−0.11 (−2.92, 2.96)	−0.20 (−1.94, 2.53)	0.33 (−1.98, 2.37)	0.43 (−1.70, 2.26)
*Unknown*	52	17	11	17	7

**Table 2:** Overall and outcome-specific distribution of the bacterial toxins genes analyzed in the study.

Characteristic	Overall, N = 325	Healthy Tissue (Controls), N = 162	Hyperplastic Polyps (HP), N = 55	Polyps/Adenoma (PA), N = 79	Adenocarcinoma (ADK), N = 29
***cif, n (%)***	32 (10%)	11 (7.1%)	7 (13%)	12 (16%)	2 (7.4%)
*Unknown*	17	7	3	5	2
***cdt, n (%)***	40 (13%)	18 (12%)	2 (3.8%)	15 (20%)	5 (19%)
*Unknown*	17	7	3	5	2
***cnf1, n (%)***	89 (29%)	42 (27%)	16 (31%)	20 (27%)	11 (41%)
*Unknown*	17	7	3	5	2
***pks+, n (%)***	117 (38%)	54 (35%)	22 (42%)	27 (36%)	14 (52%)
*Unknown*	17	7	3	5	2
***bft, n (%)***	43 (13%)	23 (14%)	6 (11%)	10 (13%)	4 (14%)
*Unknown*	6	3	1	1	1

**Table 3:** Results of univariate and multivariate logistic regression model for tissues with adenocarcinomas (ADK) of colon-rectum compared to healthy tissues (controls). Univariate odds ratios are also shown for all factors with univariate association within 10% tolerance (*p*-value < 0.1) that were included in the multivariate model and for all bacterial toxins.

	Univariate			Multivariate		
Characteristic	OR ^1^	95%CI ^1^	*p*-Value ^1^	OR ^1^	95%CI ^1^	*p*-Value ^1^
***cif,* yes vs. no**	1.05	0.16, 4.21	0.95			
***cdt,* yes vs. no**	1.73	0.53, 4.86	0.32			
***cnf1,* yes vs. no**	1.85	0.78, 4.28	0.15			
**pks+, yes vs. no**	2.01	0.88, 4.64	0.10			
***bft,* yes vs. no**	0.99	0.27, 2.85	0.98			
**Sum of aerobic toxins**	1.60	1.02, 2.54	**0.042**			
**Sum of all toxins**	1.47	0.96, 2.24	0.073			
**Polygenic Risk Score (PRS)**	1.55	1.00, 2.45	0.055	1.55	1.00, 2.45	0.055
**Age**	1.04	1.01, 1.08	**0.027**			

^1^ OR, odds ratio; CI, confidence interval; in bold, *p*-value < 0.05.

**Table 4:** Results of univariate and multivariate logistic regression model for tissues with polyps or adenomas (PA) of colon-rectum compared to healthy tissues (controls). Univariate odds ratios are also shown for all factors with univariate association within 10% tolerance (*p*-value < 0.1) that were included in the multivariate model and for all bacterial toxins.

	Univariate			Multivariate		
Characteristic	OR ^1^	95%CI ^1^	*p*-Value ^1^	OR ^1^	95%CI ^1^	*p*-Value ^1^
***cif,* yes vs. no**	2.53	1.06, 6.14	**0.036**	2.57	1.06, 6.33	**0.037**
***cdt,* yes vs. no**	1.94	0.90, 4.10	0.085			
***cnf1,* yes vs. no**	1.20	0.59, 2.35	0.61			
**pks+, yes vs. no**	1.37	0.72, 2.60	0.33			
***bft,* yes vs. no**	0.74	0.26, 1.82	0.54			
**Polygenic Risk Score (PRS)**	1.36	1.00, 1.87	0.051			
**Sex, F vs. M**	0.49	0.28, 0.85	**0.011**	0.48	0.27, 0.84	**0.011**
**BMI**	1.07	1.00, 1.15	0.068			
**Wine consumption, yes vs. no**	1.58	0.92, 2.74	0.10			

^1^ OR, odds ratio; CI, confidence interval; in bold, *p*-value < 0.05.

**Table 5:** Results of univariate and multivariate logistic regression model for tissues with hyperplastic polyps (HP) of colon-rectum compared to healthy tissues (controls). Univariate odds ratios are also shown for all factors with univariate association within 10% tolerance (*p*-value < 0.1) that were included in the multivariate model and for all bacterial toxins.

	Univariate			Multivariate		
Characteristic	OR ^1^	95%CI ^1^	*p*-Value ^1^	OR^1^	95%CI ^1^	*p*-Value ^1^
***cif,* yes vs. no**	2.04	0.71, 5.49	0.17			
***cdt,* yes vs. no**	0.30	0.05, 1.11	0.12			
***cnf1,* yes vs. no**	1.20	0.59, 2.35	0.61			
**pks+, yes vs. no**	1.37	0.72, 2.60	0.33			
***bft,* yes vs. no**	0.74	0.26, 1.82	0.54			
**Sex, F vs. M**	0.42	0.22, 0.79	**0.007**			
**BMI**	1.08	1.00, 1.16	**0.047**	1.10	1.01, 1.19	**0.026**
**Alcohol consumption, yes vs. no**	2.07	1.06, 4.02	**0.032**			
**Non-drinker, yes vs. no**	0.25	0.06, 0.74	**0.027**	0.23	0.05, 0.73	**0.026**
**Smoking**						
Yes vs. no	2.81	1.31, 6.13	**0.008**			
Ex vs. no	1.54	0.73, 3.24	0.25			
**Smoking, yes vs. no**	2.01	1.07, 3.88	**0.034**			
**Previous removal of polyps**						
Yes benign vs. yes malignant	5.79	2.10, 18.9	**0.001**	5.45	1.90, 18.3	**0.003**
No vs. yes malignant	1.31	0.49, 4.16	0.62	1.17	0.42, 3.81	0.78

^1^ OR, odds ratio; CI, confidence interval; in bold, *p*-value < 0.05.

## Data Availability

The data presented in this study are available on request from the corresponding author. The data are not publicly available due to privacy restrictions.

## Supplementary Materials

The following are available online at https://www.mdpi.com/article/10.3390/toxins13080569/s1, Table S1: Primers used in the study.
