# Supplementary material for: Association of Polygenic Risk Score and Bacterial Toxins at Screening Colonoscopy with Colorectal Cancer Progression: A Multicenter Case-Control Study
Source: Toxins (Basel). 2021 Aug 16;13(8):569. doi: 10.3390/toxins13080569 (PMC8402601; doi:10.3390/toxins13080569)
Supplement: Supplementary file 1 [file toxins-13-00569-s001.zip › toxins-1328628-SI.pdf]

Association of Polygenic Risk Score and Bacterial Toxins at Screening Colonoscopy with Colorectal Cancer Progression: A Multicenter Case-Control Study

Alfonso Piciocchi, Elena Angela Pia Germinario, Koldo Garcia Etxebarria, Silvia Rossi, Lupe Sanchez-Mete, Barbara Porowska, Vittoria Stigliano, Paolo Trentino, Andrea Oddi, Fabio Accarpio, Gian Luca Grazi, Giovanni Bruno, Massimo Bonucci, Massimo Giambenedetti, Patrizia Spigaglia, Fabrizio Barbanti, Slawomir Owczarek, Ida Luzzi, Elisabetta Delibato, Zaira Maroccia, Lorenza Nisticò, Carla Fiorentini, Mauro D'Amato, Roberta De Angelis, and Alessia Fabbri

Table S1

Gene target	Amplicon (bp)	Annealing temp (°C)	Melting temp (°C)	Primer concentration (nM)
cnf1mac1 (1)	mac1 5'-GGG GGA AGT ACA GAA GAA TTA- 3' (Biomers.net)	52	78.5±1	200
cnf1mac2 (1)	mac2 5'-TTG CCG TCC ACT CTC ACC AGT –3' (Biomers.net)	52	78.5±1	200
cdt-S2 (1)	S2 5'-GAA AAT AAA TGG AAC ACA CAT GT CCG –3' (Biomers.net)	52	80±1	200
cdt-AS2 (1)	AS2 5'-AAA TCT CCT GCA ATC ATC CAG TTA –3' (Biomers.net)	52	80±1	200
gapA2 (This study)	5'-CAG AAC ATC ATC CCG TCC-3' (Biomers.net)	52	82.5±1	200
gapA1 (This study)	5'-GTT CAG AGC GAT ACC AGC-3' (Biomers.net)	52	82.5±1	200
cif-P1 (1)	5'- AACAGATGGCAACAGACTGG - 3' (ISC Bio Basic INC)	52	80±1	200
cif-P2 (1)	5'- AGTCAATGCTTTATGCGTCAT –3' (ISC Bio Basic INC)	52	80±1	200
pks clbA1 (1)	5'-CAG ATA CAC AGA TAC CAT TCA –3' (Biomers.net)	55	86.5±1	200
pks clbA2 (1)	5'-CTA GAT TAT CCG TGG CGA TTC–3' (Biomers.net)	55	86.5±1	200
pks clbQ1 (1)	5'-TTA TCC TGT TAG CTT TCG TTC –3' (Biomers.net)	55	86.5±1	200
pks clbQ2 (1)	5'-CTT GTA TAG TTA CAC AAC TAT TTC–3' (Biomers.net)	55	86.5±1	200
b-fra R (2)	R 5'-CCA GTA TCA ACT GCA ATT TTA-3' (Biomers.net)	52	82±1	200
b-fra F (2)	F 5'-ATA GCC TTT CGA AAG RAA GAT-3' (Biomers.net)	52	82±1	200
bft 1 (3)	5'-GAC GGT GTA TGT GAT TTG TCT GAG AGA-3' (ISC Bio Basic INC)	52	79±1	200
bft 2 (3)	5'-ATC CCT AAG ATT TTA TTA TCC CAA GTA-3' (ISC Bio Basic INC)	52	79±1	200

References

Buc et al., 2013 DOI: 10.1371/journal.pone.0056964
Matsuki et al., 2002 DOI:10.1128/AEM.68.11.5445-5451.2002
Pantosti et al., 1997 PMID: 9316893
